# AI Use in Psychotherapy Teaching and Training: The Perspective of Psychotherapy Tutors Across England

**DOI:** 10.1192/bjo.2026.11273

**Published:** 2026-06-30

**Authors:** Alina Vaida, Uzair Asif

**Affiliations:** 1 CWPT, Warwick, United Kingdom; 2 CWPT, Coventry, United Kingdom

## Abstract

**Aims:**

To evaluate the impact of AI use on psychotherapy training for “core trainee” psychiatry residents as understood by the psychotherapy tutors. The psychotherapy training for residents includes theory-based teaching, courses, Balint groups, and completion of two psychotherapy cases across different modalities, all supervised by experienced psychotherapists. This includes an anonymised case summary with a formulation.

**Methods:**

A questionnaire was sent to the psychotherapy tutors via a national (England) email list enquiring about their use of AI in psychotherapy training. The data is both quantitative and qualitative, summarising emerging themes. A similar survey was done for core trainees and is reported in a separate poster.

**Results:**

16 tutors completed the questionnaire (sent to 48 tutors).

75% of tutors stated that they don’t use AI for psychotherapy training or teaching. Of the 4 tutors who use AI, their responses include using it to: brainstorm, create images, find references, summarise different models, or serve as a “thinking partner”.

Most tutors (56.3%) were unsure whether residents used AI; 18.8% said residents used it, and 25% reported they didn’t. 7 tutors commented on the residents’ use of AI, with 4 expressing concerns about its use for formulations or summary letters. Other examples of AI use included teaching planning and creating captions for recorded sessions.

When combining the “yes” and “maybe” responses, support for potential AI use was greater for teaching psychotherapy concepts than for assisting with scribing or aiding formulations (75%, 43.8%, and 43.8%, respectively).

56.3% of tutors expressed concerns about the impact on relevant learning and critical thinking, especially the reflective and emotional aspects of the psychotherapeutic work. Further concerns regarding confidentiality and misrepresentation of the cases were raised if AI was used to record or summarise information. Two tutors also expressed concerns about how patients may be using AI.

When asked how best to use AI, the most common response (37.5%) was to use it to summarise concepts or theory.

**Conclusion:**

The results demonstrated concerns regarding the impact on psychotherapy training (acquiring reflective capacity skills, emotional learning, critical thinking skills) and confidentiality, however there was support for utilising AI especially for learning or summarising psychotherapy concepts. Some concerns may be more easily addressed through governance policies (e.g. data protection), but the potential impact on experiential learning requires further discussion and guidance.

